# A Glimpse at the Anti-Phage Defenses Landscape in the Foodborne Pathogen *Salmonella enterica* subsp. *enterica* serovar Typhimurium

**DOI:** 10.3390/v15020333

**Published:** 2023-01-24

**Authors:** Cedric Woudstra, Sophie A. Granier

**Affiliations:** 1 Department of Veterinary and Animal Sciences, University of Copenhagen, Stigbøjlen 4, 1870 Frederiksberg C, Denmark; 2 Antibiotics, Biocides, Residues and Resistance Unit, Fougères Laboratory, ANSES, 35306 Fougères, France

**Keywords:** *Salmonella*, *S*. Typhimurium, anti-phage defense systems, PADLOC, mobile genetic elements, bacteriophage

## Abstract

Bacteriophages, which specifically infect and kill bacteria, are currently used as additives to control pathogens such as *Salmonella* in human food (PhageGuard S^®^) or animal feed (SalmoFREE^®^, Bafasal^®^). Indeed, salmonellosis is among the most important zoonotic foodborne illnesses. The presence of anti-phage defenses protecting bacteria against phage infection could impair phage applications aiming at reducing the burden of foodborne pathogens such as *Salmonella enterica* subsp. *enterica* serovar Typhimurium (*S*. Typhimurium) to the food industry. In this study, the landscape of *S*. Typhimurium anti-phage defenses was bioinformatically investigated in publicly available genomes using the webserver PADLOC. The primary anti-phage systems identified in *S*. Typhimurium use nucleic acid degradation and abortive infection mechanisms. Reference systems were identified on an integrative and conjugative element, a transposon, a putative integrative and mobilizable element, and prophages. Additionally, the mobile genetic elements (MGEs) containing a subset of anti-phage systems were found in the *Salmonella enterica* species. Lastly, the MGEs alone were also identified in the *Enterobacteriaceae* family. The presented diversity assessment of the anti-phage defenses and investigation of their dissemination through MGEs in *S*. Typhimurium constitute a first step towards the design of preventive measures against the spread of phage resistance that may hinder phage applications.

## 1. Introduction

Non-typhoidal *Salmonella* are zoonotic pathogenic bacteria that cause diarrhea in humans by food poisoning [1]. More than 90 million cases and 155,000 deaths worldwide each year are estimated to be due to salmonellosis [2]. Mostly responsible are *Salmonella enterica* subsp. *enterica* serovar Enteritidis and serovar Typhimurium [3]. The USDA estimated that foodborne illnesses caused by non-typhoidal *Salmonella* cost the USA USD 4 billion in 2018 [4]. *Salmonella* infections commonly occur after consumption of contaminated poultry products [5]. Therefore, a variety of control measures, such as biosecurity, eradication, or vaccination, have been implemented in poultry and egg production to prevent *Salmonella* outbreaks [6]. Recently, there has been interest in using bacteriophage therapy to control *Salmonella* in live poultry [7]. Bacteriophages (phages) are viruses that specifically infect and kill bacteria. Owing to the antimicrobial crisis, they have received increasing attention as an alternative to chemical antibiotics [8]. They are used therapeutically in last-resort treatment of multidrug-resistant bacterial infections (e.g., due to MRSA [9]) and as biocontrol agents in human food and animal feed production [10]. In various parts of the world [11], phages are currently approved to be used as additives to control *Salmonella* in human food (PhageGuard S^®^, [12]) or animal feed (SalmoFREE^®^, Bafasal^®^ [13,14,15]). Bacteria, however, can become resistant to phages [16]. Like antibiotic resistance, phage resistance arises either from mutations in the bacterial phage receptor or through other phage resistance mechanisms [17]. Lately, a new plethora of anti-phage defense systems protecting bacteria against phage infections have been discovered and characterized [18,19,20]. Furthermore, they have been found to be associated with mobile genetic elements (MGEs) [21]. Thus, the increase in phage application as antimicrobials could select for bacteria carrying anti-phage defense systems and trigger their dissemination through MGEs. This would lead to the development of bacterial populations resistant to phage treatment, as occurred previously with the emergence of antibiotic resistances [22]. As phages are already commercially available for control of *Salmonella*, the evaluation of the anti-phage defense landscape in *Salmonella* is crucial to develop future preventive measures against the possible spread of phage resistance in this foodborne pathogen.

Therefore, the aim of this study was to assess the anti-phage defense content of *Salmonella enterica* subsp. *enterica* serovar Typhimurium (*S*. Typhimurium) and their mobility between different bacterial species. This study reports that nucleic acid degradation and abortive infection anti-phage defenses were the most present mechanisms of defense. They were observed within different types of MGEs that could be also found in other serovars of *Salmonella* such as *S.* Enteritidis.

## 2. Materials and Methods

A diagram of the analysis performed and the results obtained are available as graphical abstract.

### 2.1. Detection of Anti-Phage Defense Systems Using the Webserver PADLOC

Anti-phage defense systems from *S*. Typhimurium were searched in the PADLOC webserver’s precomputed results of the RefSeq v209 database, available online from https://padloc.otago.ac.nz/padloc/refseq/ (accessed on 1 September 2022) [23]. Bulk data were extracted based on genomes’ GCF accession numbers. A total of 1564 genome sequences were analyzed. Anti-phage defense systems found within the 1564 genome sequences were sorted and their proportions calculated.

### 2.2. Reference Sequences of S. Typhimurium Anti-Phage Defense Systems

To investigate the diversity of anti-phage defense system types, within each detected system type a reference nucleotide sequence was chosen arbitrarily. Restriction modification (RM) systems and clustered regularly interspaced short palindromic repeats (CRISPR)/CRISPR-associated protein (Cas) were not investigated, as work on these two systems has already been published previously [24,25]. *S*. Typhimurium strain 157 A was chosen as a reference for containing the BREX type I anti-phage defense system (contig accession number NZ_JACYBM010000004.1). Strain 5725 was chosen for containing the retron type II-A, Gabija, and ietAS systems (contig accession number NZ_JABBKQ010000016.1). Strain AR-0408 was chosen for containing the PARIS system (accession number NZ_CP044198). Strain ST45 was chosen for containing both the Kiwa and AbiU systems (CP50753.1). Strain 1387 A was chosen for representing the AbiD system (contig accession number NZ_JACXZY010000007.1).

### 2.3. Presence of Reference Anti-phage Defense Systems in S. Typhimurium

Nucleotide sequences from each reference anti-phage defense system were searched using BLASTn (accessed on 12 September 2022) [26] with a 90% identity and 90% coverage cut-off against the NCBI refseq genomes database including only *S*. Typhimurium taxid:90371 (24461 sequences [26], accessed on 12 September 2022). The presence of the reference anti-phage defense systems is reported in results Section 3.2.

### 2.4. Presence of Reference Anti-phage Defense Systems Co-occurring with Integrases

To explore the mobility of the identified anti-phage defense systems, each reference system chosen in Table 1 was searched for integrase related genes in their vicinity. Following, to evaluate the co-occurrence of integrases and reference defense systems, their nucleotide sequences were concatenated and searched using BLASTn against the NCBI refseq genomes of *S*. Typhimurium taxid:90371 database (24461 sequences [26], accessed on 12 September 2022). The number of returned hits with a 90% identity and 90% coverage cut-off from the integrase and the defense systems were then compared to the results of results Section 3.2, where only the defense systems were searched, and compiled in results Section 3.3.

### 2.5. Presence of Reference Anti-phage Defense Systems and Their Integrases in Bacterial Species Other than Salmonella Typhimurium

To detect mobility of the reference anti-phage systems, allowing them to integrate into the genomes of other bacterial species, the nucleotide sequence of the reference systems together with their associated integrases were compared with a 90% identity and 90% coverage cut-off against the NCBI refseq all complete genomes database excluding *S*. Typhimurium taxid:90371 (77327 sequences [26], accessed on 12 September 2022). Their proportion in comparison to the non-*S*. Typhimurium refseq genomes is reported in results Section 3.4.

### 2.6. Presence of Integrases Alone from Reference Anti-phage Defense Systems in Non-Salmonella Typhimurium Bacterial Species

To investigate if the mobile elements alone without the anti-phage defense systems could be found outside of *S*. Typhimurium, the nucleotide sequences of the integrases only were used in a BLASTn search against the NCBI refseq genome database excluding *S*. Typhimurium taxid:90371 (77327 sequences [26], accessed on 12/09/2022). The number of hits results with a 90% identity and 90% coverage cut-off were recorded and the percentage calculated in comparison with the non-*S*. Typhimurium genome sequences. Results are summarized in results Section 3.5.

## 3. Results

### 3.1. S. Typhimurium Uses Mainly Nucleic Acid Degradation and Abortive Infection Anti-Phage Defense System Types

**Figure 1:**
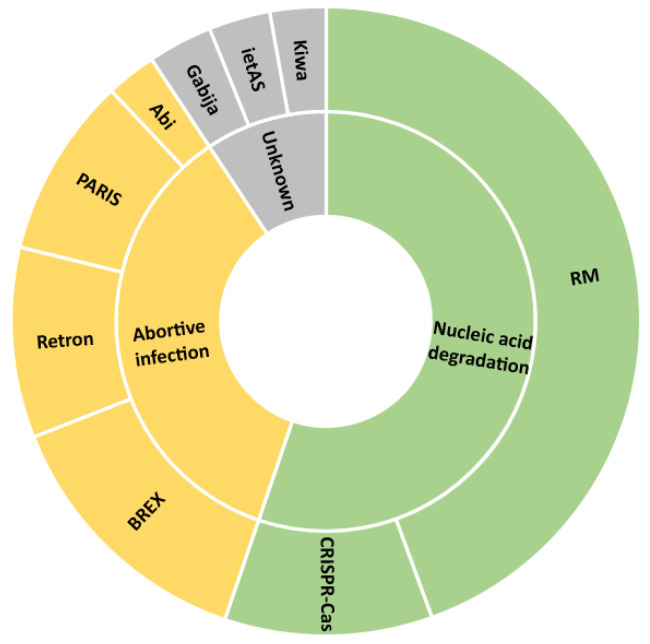
Presence of anti-phage defense systems in *S*. Typhimurium.

Within the 1564 refseq genomes present in the PADLOC database anti-phage defense systems were identified in 1563 of them (Table S1). Nucleic acids degradation systems such as RM systems, with an average of four RM per strain (type I, II, II-G, III, and IV), as well as CRISPR-Cas (mainly type I-E), were detected predominantly (Figure 1, Table S1). Abortive infection defense systems such as the bacteriophage exclusion (BREX, mainly type I), Retron (type I-C, II-A, V, XII), the phage anti-restriction-induced system (PARIS), and abortive infection Abi (Abi-D, -E, -L, -Q, -U) were also found repeatedly (Figure 1, Table S1). Defense systems Gabija, ietAS, and Kiwa, which use uncharacterized mechanisms of defense, were also identified, to a lower extent (Figure 1, Table S1). Other systems were also detected with a frequency below 10% of the analyzed genomes (Table S1).

The inner circle represents the mechanism type of anti-phage defense systems as categorized by Nitzan Tal and Rotem Sorek [27]. The outside circle represents the name of the defense systems. Only defense systems identified in >10% of the genome sequences are represented. RM: restriction modification; CRISPR: clustered regularly interspaced short palindromic repeats; BREX: bacteriophage exclusion; PARIS: phage anti-restriction-induced system; Abi: abortive infection.

### 3.2. S. Typhimurium Harbors Genetic Diversity within Each Type of Anti-phage Defense System

To test for possible genetic diversity within the identified defense systems, a reference sequence from each system type was searched against the NCBI refseq genomes database (Table 1).

BREX system type I was identified by PADLOC as present in 90% of the *S*. Typhimurium sequences within the refseq database v209. Here, using the nucleotide sequence from *S*. Typhimurium strain 157 A as reference, 6% of all genomes available in the refseq NCBI database were found to encompass this exact system. Seemingly, anti-phage retron type II-A (present in reference strain *S*. Typhimurium 5725) and PARIS (in reference strain AR-0408) defense systems, which were found in 89% and 86% of refseq v209 with PADLOC, were detected in 6% of all *S*. Typhimurium genomes available in the refseq NCBI database, respectively. Systems Gabija, ietAS, and Kiwa were found by PADLOC to be present in 31%, 29%, and 26% of all *S*. Typhimurium genomes, respectively. Yet, these systems were present only in 2% of all genomes from NCBI refseq database. Regarding abortive infection Abi defense type, AbiD and AbiU were present in 11% of PADLOC results, yet were present in 1% and 0.3% of the refseq NCBI genomes, respectively. These results indicate the presence of genetic diversity within all the anti-phage defense systems of *S*. Typhimurium presently detected by PADLOC.

### 3.3. S. Typhimurium Anti-Phage Defense Systems Can Be Associated with Diverse Integrases

The association between anti-phage defense systems and integrases, which are indicative of genetic mobility, can shed light on the presence of these defense systems within MGEs (Table 2).

The search revealed that different types of MGEs are associated with the investigated reference defense systems, such as the *Salmonella* genomic island 1 integrative and conjugative element, prophages, a transposon and a putative integrative and mobilizable element. In addition, defense systems BREX type I, PARIS, Gabija, ietAS and AbiD were mostly found associated with the respective reference integrases (100%, 93%, 95%, 91% and 91% respectively). This suggests that these reference defense systems move using one integrase from one MGE. On the contrary, the reference defense systems from retron type II-A and Kiwa were poorly associated with the reference integrase (5% and 13%), while AbiU was associated in 61% with the reference integrase, indicating that they can be found associated with potentially other integrases on different MGEs.

### 3.4. S. Typhimurium Integrases Associated with Anti-phage Defense Systems Are found Mainly in Salmonella Enterica Species

Considering that *S*. Typhimurium contains anti-phage defense systems on MGEs, they may disseminate to other bacteria. To investigate this possibility the nucleotide sequences of each integrase concatenated with their respective defense systems were searched in the NCBI refseq complete genomes database excluding *S*. Typhimurium (Table 3).

Anti-phage defense systems and their integrases were found in less than 1% of non-*S*. Typhimurium genome sequences. In total, *Salmonella enterica* was the main species harboring similar anti-phage defense systems (89%), followed by *Proteus mirabilis* (6%), *Escherichia coli* (4%), and *Vibrio sp*. (1%). This indicates that the reference defense systems disseminate mainly within the same bacterial species.

### 3.5. Integrases Alone from Reference Anti-phage Defense Systems Are Found Mainly in Salmonella and in Other g-Proteobacteria Species

Anti-phage defense systems and their integrases identified in *S*. Typhimurium can also be found in *Salmonella* non-Typhimurium. To evaluate if the MGEs identified to contain the reference anti-phage defense systems could be found in other bacterial species, their integrases alone were searched in non-*S*. Typhimurium bacteria (Table 4).

A relatively higher hits count was found to match with the search for integrases alone in comparison to the search for integrases together with their anti-phage defense systems in the NCBI refseq genomes database, excluding *S*. Typhimurium. The bacteria species found to contain these integrases all belong to the g-proteobacteria, and primarily to the *Enterobacteriaceae* family. They were mainly from *Salmonella enterica* followed by *Escherichia*, *Enterobacter*, *Klebsiella* and *Citrobacter* species. *Morganellaceae* (from the *Proteus* genus) and *Shewanellaceae* (from the *Shewanella* genus) family were also detected, to a lower extent.

## 4. Discussion

### 4.1. S. Typhimurium and its Phage Defenses

Here, *S*. Typhimurium was investigated for its anti-phage defense systems content. The anti-phage systems detection was based on the PADLOC webserver [28,29], which uses the hidden Markov model’s (HMM) protein similarities prediction [28]. The webserver is designed to detect and classify a large range of anti-phage defense systems [29]. With a unique exception, PADLOC identified in each *S*. Typhimurium strain anti-phage defense systems from the genome sequences present in the refseq v209. This result was expected since bacteria face phage predation in environments where they are co-occurring. It might also be hypothesized that, for the unique genome of *S*. Typimurium in the database with no phage defense trait, an incorrect annotation of this genome is involved.

However, from the information stored in the PADLOC webserver, the genetic diversity within each anti-phage defense system type cannot be investigated, as PADLOC uses HMM protein similarities to detect anti-phage defenses. In this study, the genetic diversity within each anti-phage defense system type was probed based on defining one reference sequence per defense system. Though it is to be expected that the results obtained would differ depending on the chosen reference systems, we decided to arbitrarily pick one reference per system as a first screening of the presence of genetic diversity. A further detailed study of the defense systems phylogenetic diversity would aid in understanding how *S*. Typhimurium acquires and disperses them.

Nucleic acid degradation and abortive infection were the two most prominent anti-phage mechanisms detected (Figure 1). Anti-phage nucleic acid degradation systems (e.g., CRISPR-Cas, RM systems) are based on proteins that can identify invading genetic elements, here phages, and differentiate them from the bacterium’s own DNA [30]. In this case, the genetic invader is destroyed, and the bacterium survives. Moreover, abortive infection systems (e.g., Abi, BREX systems) are expressed when the bacterium senses that a viral infection is already in progress, and triggers its own death. Therefore, the infected bacterium dies altruistically, protecting the overall bacterial population [31]. Both types of mechanism have been widely studied and have been shown to efficiently protect bacteria against phage predation [32,33]. Therefore, it is not surprising to find these mechanisms as part of the main arsenal of *S*. Typhimurium against phage infection. In addition, more recently identified defense systems have also been detected in this study in *S*. Typhimurium (Gabija, ietAS, Kiwa [34,35]). Gabija has been recently partially characterized to be a nucleotide-sensing endonuclease, while the mechanism of action of ietAS and Kiwa are still awaiting to be described. In addition, other defense systems have been detected only rarely (Table S1) and were not analyzed further within the present study. In the meantime, phages are able to acquire new counter-attacks to keep up in this armed race [36]. Phages infecting *Salmonella* can overcome anti-phage defenses based on nucleic acid degradation by modifying their thymidine nucleotide bases [37,38]. Abortive infection can also be neutralized through mutations or deletion of specific phage regions that are recognized by the bacterial immunity system [31,39].

### 4.2. S. Typhimurium Carries its Phage Defenses on Mobile Genetic Elements

In this study, we also investigated the presence of anti-phage defenses on MGE in *S*. Typhimurium. As demonstrated by earlier work, anti-phage defense systems can be shared among bacteria as they are often associated with MGEs [21,40]. In addition, it is common for MGEs to cross species barriers and disseminate in other bacterial species, which can, for example, result in the spread of antimicrobial resistance [41]. For example, plasmids have been shown to exchange DNA beyond the genus (and phylum) barrier [42]. Here, prophages, the *Salmonella* genomic island 1 integrative and conjugative element [43], a transposon, a putative integrative and mobilizable element [44] have been found to encompass systems defending *S*. Typhimurium against phage predation. These MGEs were also found at a low frequency within *S. enterica* and other related bacterial families, indicating that the dissemination of anti-phage systems beyond *S*. Typhimurium is possible. It is expected that further MGEs containing anti-phage defense systems could be uncovered in *S*. Typhimurium as the diversity of integrative and conjugative elements and integrative and mobilizable elements and their role in horizontal gene transfer of resistance towards chemical and biological antimicrobials is just starting to be recognized [44,45,46]. In addition, it is established that antibiotic treatment increases the frequency of horizontal gene transfer by conjugation [47] and defense systems can cluster on MGEs that carry already antibiotic resistance, such as the integrative and conjugative element SXT [48]. Therefore, the use of antibiotic substances could play a role in the concomitant spread of anti-phage defense systems together with other antimicrobial resistance traits (i.e., towards chemical antimicrobials).

### 4.3. Implications of the Mobility of Anti-phage Defense Systems in S. Typhimurium for Using Bacteriophages as Food and Feed Additives

Bacteria have developed a multitude of defense systems to counter bacteriophage infection, which are the focus of intense and prolific recent research [27,49,50,51,52]. By sharing these defenses via MGEs, bacteria can acquire phage resistance. Therefore, the commercial use of bacteriophages as biocontrol agents for protection of food and feed against *Salmonella* [12,15] could be impaired. The phages contained within the currently commercially available phage products have been selected to be active against a selection of *Salmonella* strains. Therefore, it can be anticipated that these phages are able to overcome *Salmonella* anti-phage defenses. Thus, by increasing the phage selective pressure on *Salmonella*, it could be expected that strains protected by different effective anti-phage defense systems could be selected, and their anti-phage systems disseminated and acquired more frequently. This will lead to the development of bacterial populations resistant to phage treatment, as it occurred previously with the emergence of antibiotic resistances. To deal with the dissemination of phage resistance, the food industry would have to keep their phage products up to date by replacing phages that would have become inefficient. This strategy will require monitoring the apparition of phage resistance within *Salmonella* gender through either whole genome sequencing [53], or phage typing assay [54]. Another option would be to engineer phages to increase their killing efficiency. Nonetheless, while methods to genetically engineer phages are readily available [55,56], to the best of our knowledge, the use of genetically modified phage has not been approved by any authorities worldwide [9].

### 4.4. Are Phage Defense Systems Possibly Involved in Salmonella Epidemiological Success?

Readers might have noticed that this study carefully avoid using the term “prevalence” as the primary genome database used reflects only *S*. Typhimurium sequences available in the refseq v209. We might assume that the genome sequences mostly represented are those of public health concern that were collected by medical doctors, veterinarians, clinical microbiologists, and outbreaks within the food industry. Therefore, the proportions that we calculated in this study are not representative of any epidemiological situation. Nonetheless, this study still highlights a high frequency of detection of phage defense systems. This raises questions about the evolutionary advantage of epidemic strains that might harbor efficient phage defense systems. Authors have from time to time evocated that *Salmonella* persistence in food processing plants might be driven by a specific ability to resist desiccation [57] or biocide substances [58]; our findings might add to the list anti-phage defense systems. To determine if phage defense mechanisms are evolutionary advantageous for *Salmonella* in food processing or farm environments, a new broader investigation should be performed over a larger genome database including the successful clones of *Salmonella* that emerged worldwide over the past decades, such as *S*. Kentucky ST198 [59] or the monophasic variant of *S*. Typhimurium [60] or more recently *S.* Infantis [61].

## 5. Conclusions

This study has explored the anti-phage defense content within *S*. Typhimurium and their dissemination through MGEs. Therefore, it constitutes an important first step towards the understanding of such defenses to design future preventive measures against the spread of these resistance mechanisms, which could impair phage applications in the food industry.

## Figures and Tables

**Table 1:** Presence of reference defense systems in *S*. Typhimurium.

Anti-Phage Defense System	Presence within PADLOC Refseq v209 * (*n* = 1564)	Accession Number of *S*. Typhimurium Strain Harboring ref. Defense Systems	Presence of ref. Defense Systems in Refseq Genomes * (NCBI; *n* = 24,461)
BREX type I	90%	NZ_JACYBM010000004.1	6%
retron type II-A	89%	NZ_JABBKQ010000016.1	6%
PARIS	86%	NZ_CP044198.1	6%
Gabija	31%	NZ_JABBKQ010000016.1	2%
ietAS	29%	NZ_JABBKQ010000016.1	2%
Kiwa	26%	NZ_CP050753.1	2%
AbiD	11%	NZ_JACXZY010000007.1	1%
AbiU	11%	NZ_CP007523.1	0.3%

* *S*. Typhimurium taxid:90371. BREX: bacteriophage exclusion; PARIS: phage anti-restriction-induced system; Abi: abortive infection.

**Table 2:** Association between reference defense systems and their integrases.

	Ref. Anti-Phage System Accession Number		Refseq Genomes *
Int. + System		MGE Type	Percentage Compared to Positives in Table 1
Int. + BREX type I	NZ_JACYBM010000004.1	probable IME	100%
Int. + retron II-A	NZ_JABBKQ010000016.1	ICE	5%
Int. + PARIS	NZ_CP044198.1	prophage	93%
Int. + Gabija	NZ_JABBKQ010000016.1	ICE	95%
Int. + ietAS	NZ_JABBKQ010000016.1	ICE	91%
Int. + Kiwa	NZ_CP050753.1	prophage	13%
Int. + AbiD	NZ_JACXZY010000007.1	transposon	91%
Int. + AbiU	NZ_CP007523.1	prophage	61%

* *S*. Typhimurium taxid:90371. MGE: mobile genetic elements. Int: integrase. ICE: integrative and conjugative element. IME: integrative and mobilizable element. BREX: bacteriophage exclusion; PARIS: phage anti-restriction-induced system; Abi: abortive infection.

**Table 3:** Presence of both integrases and reference anti-phage defense systems in non-*S*. Typhimurium.

MGE-int. + Anti-Phage Defense System	Accession Number	BLASTn Count	Presence in Refseq Genomes * (*n* = 77,327)	Detected Species (% of Hits)
Int. + BREX type I	NZ_JACYBM010000004.1	159	0.2%	*Salmonella enterica* 100%
Int. + retron II-A	NZ_JABBKQ010000016.1	6	0.0%	*Salmonella enterica* 100%
Int. + PARIS	NZ_CP044198.1	172	0.2%	*Salmonella enterica* 99%, *Enterobacter* 1%
Int. + Kiwa	NZ_CP050753.1	115	0.1%	*Salmonella enterica* 100%
Int. + Gabija	NZ_JABBKQ010000016.1	66	0.1%	*Salmonella enterica* 56%, *Proteus* 33%, *Vibrio* 5%, other 6%
Int. + ietAS	NZ_JABBKQ010000016.1	62	0.1%	*Salmonella enterica* 56%, *Proteus* 34%, *Vibrio* 5%, other 5%
Int. + AbiD	NZ_JACXZY010000007.1	94	0.1%	*Salmonella enterica* 78%, *Escherichia coli* 22%
Int. + AbiU	NZ_CP007523.1	16	0.0%	*Salmonella enterica* 100%

* excluding *S*. Typhimurium taxid:90371. MGE: mobile genetic element. Int: integrase. BREX: bacteriophage exclusion; PARIS: phage anti-restriction-induced system; Abi: abortive infection.

**Table 4:** Presence of associated anti-phage defense system integrases alone in non-*S*. Typhimurium bacteria.

MGE-Int.	Accession Number	BLASTn Count	Presence in Refseq Genomes * (*n* = 77,327)	Detected Species (% of Hits)
Int._BREX type I_	NZ_JACYBM010000004.1	324	0.4%	Other *Salmonella* 64%, *Escherichia* 24%, *Klebsiella* 9%, other 3%
Int._retron II-A_	NZ_JABBKQ010000016.1	70	0.1%	Other *Salmonella* 63%, *Proteus* 24%, *Klebsiella* 4%, *Shewanella* 3%, other 6%
Int._PARIS_	NZ_CP044198.1	361	0.5%	Other *Salmonella* 54%, *Klebsiella* 16%, *Escherichia* 15%, *Enterobacter* 11%, other 4%
Int._Gabija_	NZ_CP050753.1	806	1.0%	Other *Salmonella* 89%, *Enterobacter* 8%, *Citrobacter* 3%
Int._ietAS_	NZ_JABBKQ010000016.1	70	0.1%	Other *Salmonella* 63%, *Proteus* 24%, *Klebsiella* 4%, *Shewanella* 3%, other 6%
Int._Kiwa_	NZ_JABBKQ010000016.1	70	0.1%	Other *Salmonella* 63%, *Proteus* 24%, *Klebsiella* 4%, *Shewanella* 3%, other 6%
Int._AbiD_	NZ_JACXZY010000007.1	356	0.5%	*Escherichia* 75%, other *Salmonella* 21%, *Shigella* 4%
Int._AbiU_	NZ_CP007523.1	263	0.3%	Other *Salmonella* 100%

* excluding *S*. Typhimurium taxid:90371. MGE: mobile genetic element. Int: integrase. BREX: bacteriophage exclusion; PARIS: phage anti-restriction-induced system; Abi: abortive infection.

## Data Availability

The refseq v209 database used by PADLOC to search within *S*. Typhimurium for anti-phage defense systems is available online from https://padloc.otago.ac.nz/padloc/refseq/ (accessed on 1 September 2022) [23].

## Supplementary Materials

The following supporting information can be downloaded at: https://www.mdpi.com/article/10.3390/v15020333/s1, Table S1: *S*. Typhimurium PADLOC raw results.
